# Photophysics in Biomembranes: Computational Insight into the Interaction between Lipid Bilayers and Chromophores

**DOI:** 10.1021/acs.accounts.4c00153

**Published:** 2024-08-06

**Authors:** S. Osella, S. Knippenberg

**Affiliations:** † Chemical and Biological Systems Simulation Lab, Centre of New Technologies, University of Warsaw, Banacha 2C, 02-097 Warsaw, Poland; ‡ Theory Lab, Hasselt University, Agoralaan Building D, 3590 Diepenbeek, Belgium

## Abstract

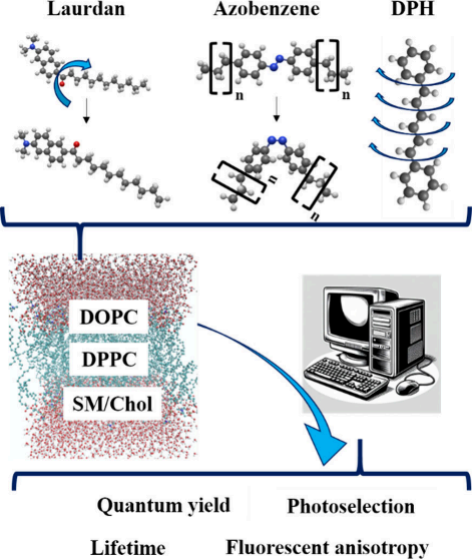

Light is ubiquitously available to probe the structure and dynamics of biomolecules and biological tissues. Generally, this cannot be done directly with visible light, because of the absence of absorption by those biomolecules. This problem can be overcome by incorporating organic molecules (chromophores) that show an optical response in the vicinity of those biomolecules. Since those optical properties are strongly dependent on the chromophore’s environment, time-resolved spectroscopic studies can provide a wealth of information on biosystems at the molecular scale in a nondestructive way. In this work, we give an overview on the multiscale computational strategy developed by us in the last eight years and prove that theoretical studies and simulations are needed to explain, guide, and predict observations in fluorescence experiments. As we challenge the accepted views on existing probes, we discover unexplored abilities that can discriminate surrounding lipid bilayers and their temperature-dependent as well as solvent-dependent properties. We focus on three archetypal chromophores: diphenylhexatriene (DPH), Laurdan, and azobenzene. Our method shows that conformational changes should not be neglected for the prototype rod-shaped molecule DPH. They determine its position and orientation in a liquid-ordered (Lo) sphingomyelin/cholesterol (SM/Chol) bilayer and are responsible for a strong differentiation of its absorption spectra and fluorescence decay times in dioleoylphosphatidylcholine (DOPC) and dipalmitoylphosphatidylcholine (DPPC) membranes, which are at room temperature in liquid-disordered (Ld) and solid-gel (So) phases, respectively. Thanks to its pronounced first excited state dipole moment, Laurdan has long been known as a solvatochromic probe. Since this molecule has however two conformers, we prove that they exhibit different properties in different lipid membrane phases. We see that the two conformers are only blocked in one phase but not in another. Supported by fluorescence anisotropy decay simulations, Laurdan can therefore be regarded as a molecular rotor. Finally, the conformational versatility of azobenzene in saturated Ld lipid bilayers is simulated, along with its photoisomerization pathways. By means of nonadiabatic QM/MM surface hopping analyses (QM/MM-SH), a dual mechanism is found with a torsional mechanism and a slow conversion for trans-to-cis. For cis-to-trans, simulations show a much higher quantum yield and a so-called “pedal-like” mechanism. The differences are related to the different potential energy surfaces as well as the interactions with the surrounding alkyl chains. When tails of increased length are attached to this probe, cis is pushed toward the polar surface, while trans is pulled toward the center of the membrane.

## Key References

- OsellaS.; PaloncýováM.; SahiM.; KnippenbergS. “Influence of membrane phase on the optical properties of DPH”, article on invitation for special issue on ‘Membrane Structure and Function’, Molecules2020, 25, 426432957614 10.3390/molecules25184264PMC7570797.^1^ We applied our computational methodology to study the molecular rotor ability of DPH embedded in DOPC.
- OsellaS.; KnippenbergS. “Laurdan as Molecular Rotor in Biological Environments”, ACS Appl. Bio. Mater.. 2019, 2, 576910.1021/acsabm.9b0078935021570.^2^ With our methodology, we observed, for the first time, the ability of Laurdan to behave like a molecular rotor in a solid gel phase.
- KnippenbergS.; OsellaS. “Push/pull effect as driving force for different optical response of azobenzene in a biological environment”, J. Phys. Chem. C2020, 124, 8310.^3^ We observed how different alkyl chain lengths are responsible for the different position and orientation of azobenzene in different membranes, resulting in different optical response.
- OsellaS.; GrannucciG.; PersicoM.; KnippenbergS. “Dual photoisomerization mechanism of azobenzene embedded in a lipid membrane”, J. Mater. Chem. B2023, 11, 251836852914 10.1039/d2tb02767d.^4^ We performed surface hopping AIMD on azobenezene embedded in DOPC, resulting in different mechanisms of photoisomerization which depend on the starting isomer.

## Introduction

Light-matter interaction is one of the most intriguing and fascinating research topic that gained strong interest in the past decade, especially when soft matter is considered.^5,6^ To be able to detect the nature, structure, and properties of soft matter with light will result in a tremendous benefit not only in the basic research field, but also—and perhaps most importantly—for its biochemical and medical applications. Lipid membranes, which exhibit in their complexity a wealth of vital functions, do not only preserve the cell from outer environments, they also play an important role for the transport of ions, nutrients, or even drugs within the cell. To carry out their essential functions, membranes must exhibit a high level of fluidity. This characteristic is influenced by both temperature and the membrane’s composition. The fluidity can change based on the type of fatty acids in the membrane and the presence of various elements like sphingomyelin and cholesterol. Research has shown a clear link between membrane fluidity and the emergence of various diseases.^7−10^ Notably, higher cholesterol levels reduce membrane fluidity, disrupt the structure of membrane nanodomains and microdomains, and support the concept of lipid rafts,^11^ which are microdomains characterized by a lower lateral mobility of its components and which could modulate cellular processes like signal transduction and endocytosis.

The presence of distinct domains within membranes is indicated by their varying softness, resulting from differences in fluidity. This heterogeneity may play a role in the spread of metastatic tumors.^12^ Therefore, understanding the characteristics of membrane fluidity is crucial to understand the involved processes regulated by membrane properties.^13^ Many studies have investigated the link between a dysregulated lipidome and cancer progression. However, there is still a substantial need to establish molecular mechanisms that connect lipids to cellular health. To thoroughly understand the biological functions of cell membranes, it is essential to have detailed knowledge of lipid organization and how they contribute to the physicochemical properties of cell membranes. However, compared to other biomolecular systems, our knowledge of membrane structure and organization is limited, because membranes are dynamic, heterogeneous, and relatively disordered.

Despite this strong effort, deep fundamental questions are still unanswered. In particular, these questions relate to how the changes in the configurations of the lipids affect the health of the tissue. Furthermore, the relationship between the composition of cell membranes and the mechanisms that regulate membrane structure and organization remains unclear. To tackle this issue, computational methods offer crucial insights into the molecular mechanisms of lateral lipid diffusion across various time and length scales. Moreover, molecular dynamics (MD) simulations have been widely used to study the local structure, dynamics, and physical properties of model lipid bilayers in different phases.^14,15^ With the rise in computational power and advancements in both classical and quantum chemical methods, the scale of investigated molecular systems has significantly expanded. Enhanced sampling techniques, such as umbrella sampling and metadynamics,^16^ have been developed. More recently, machine learning force field simulations have been introduced, further advancing the field.^17^ Biological targets can now be investigated and divided into regions, which are approached differently, with regard to the function of the intended phenomena.

Membranes can be classified in three classes: the liquid disordered (Ld), the gel (So), and the liquid ordered (Lo) phases.^18^ Membranes can be composed of single fatty acids or a combination of these with sphingomyelin (SM) and cholesterol (Chol).^19,20^ The various components of the membrane determine its phase. Membranes that are currently under investigation do often only consist of one type of lipid (such as dioleoylphosphatidylcholine [DOPC], dipalmitoylphosphatidylcholine [DPPC], or distearoylphosphatidylcholine [DSPC]), or can be based on two and three components (i.e., DOPC/SM/Chol, DOPC/DPPC). The different ratio between the components is known to lead to phases with different properties. These properties depend on the characteristics of the corresponding lipids, such as (i) lipid structure, (ii) lateral diffusion coefficient, (iii) order parameter of the alkyl chains, and (iv) temperature. The overall combination of membrane components results in a vast array of possible phase compositions. Therefore, techniques capable of characterizing and determining the different phases and, potentially, the lateral organization of the membrane are needed.

Before building and discussing the effect of realistic biological environments which incorporates all these components and facets,^17^ the effect of each separate one must be determined. Studies on lipid membranes consisting of one lipid which represent monophase systems have therefore been of utmost importance to get insight into the absorption and fluorescence spectra of probe molecules.

Probes used to label the lipid components of a membrane can highlight specific phases by selectively partitioning into different membrane domains. Some probes can even identify local differences in the surrounding membrane due to their inherent properties. Fluorescent probes are typically classified into three main categories: (i) probes that label the lipid components of the membrane, (ii) probes that selectively partition into a specific phase, and (iii) probes that can distinguish membrane phases based on their environmental sensitivity.

The latter category is the newest and most intriguing because these probes can detect membrane phases by changing their emission color, intensity, or lifetime. The appeal of these dyes lies in their ability to modify their spectroscopic properties in response to environmental changes such as viscosity, hydration, and polarity. Within this class, there are two groups: solvatochromic probes and molecular rotors. Solvatochromic probes alter their absorption properties in response to changes in the polarity of their environment, showing significant variations in their dipole moment upon excitation. Consequently, these probes’ interactions with their surroundings modify the energetics of their electronic transitions, leading to shifts in the absorption and emission peaks. Hydrogen bonding and dipole–dipole interactions between these probes and their environment influence the energies of the excited states, and induce a shift of the maxima of their absorption as well as emission spectra. The 6-dodecanoyl-2-dimethylamine-naphthalene (Laurdan), is the most known and studied probe for the detection of membrane potential changes.^21^ On the other hand, the fluorescence lifetime of a molecular rotor changes along with the frictional resistance to the probe’s rotational and translational motion. This is quantified through the microviscosity and is known to be the reciprocal to fluidity. Consequently, significant variations in fluorescence quantum yield indicate how easily the probe can rotate within different membrane domains.

In this Account, we show how our computational protocol has developed over the years to study fluorescent probes in different lipid membranes; it is not only able to explain experimental results on commonly used probes on model membranes, but we will also show its strength in prediction power to challenge the accepted view on existing probes. We selected three archetypal probes, which are representative of different families: (1) diphenylhexatriene (DPH), as a chromophore; (2) Laurdan, as a molecular rotor; and (3) azobenzene, as a novel photoswitchable probe for phase detection. In addition, we suggest how computation can give additional insight into using these probes with different purposes.

### Multiscale Computational Strategy

**Figure 1:**
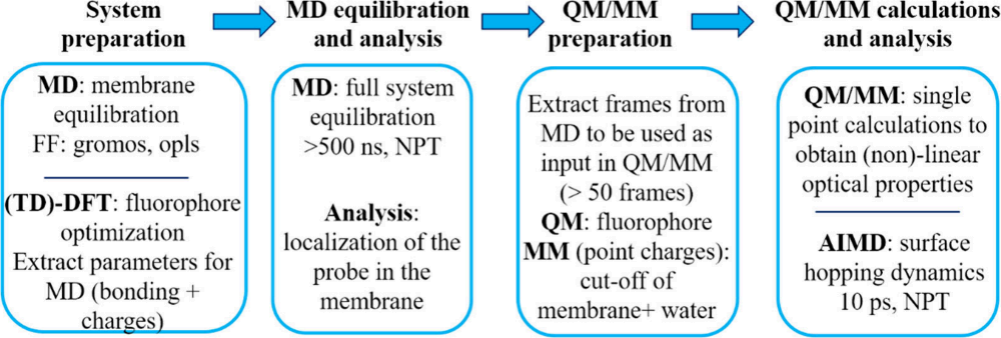
Computational protocol workflow developed to study the optical properties of fluorophores embedded in lipid membranes.

Over the past decade, we have developed a novel computational protocol to study the optical properties of organic fluorophores in a complex biological environment, such as a model membrane. The overall workflow is presented in Figure 1.

The preparation of the system consists in the equilibration of the membrane (or mixture) with classical MD simulations, to obtain a reliable membrane description. Strong attention must be paid to the parametrization of important geometrical parameters of the probes (such as angles and torsions) and partial charges to be used as input for classical molecular dynamic (MD) simulations. Hence, (TD)-DFT geometry optimization are performed to obtain the requested parameters and the probe’s transition dipole moment. Depending on the investigated properties, the MD simulations can be performed either in the ground state or in the excited state, and the proper bonding and nonbonding parameters can be considered as input. These force-field parameters have been refined and validated against ab initio methods like Møller–Plesset (MP2) for the ground state and EOM-CCSD or ADC(2) for the excited state. Next, the following MD protocol is envisaged. First, a long MD in the ground state is performed for at least 500 ns, in order to obtain a fully equilibrated system (probe + membrane) using either OPLS or GROMOS force fields. The membrane is periodic along the *x*- and *y*-directions; the *z*-axis is chosen normal to the membrane plane. From these periodic MD simulations, we can extract the orientation, position, and localization of the probe into the membrane (i.e., considering the transition dipole moment orientation). Then, from the equilibrated MD, uncorrelated frames are extracted (e.g., every 10 ns) and on each of these we performed QM/MM calculations on the absorption or emission spectra at the TD-DFT level of theory. QM/MM calculations in both ground and excited states are performed with the electrostatic embedding method, where the environment (membrane and water molecules) are considered as point charges, to account for the effect of the anisotropy of the environment over the probe’s optical response. If the excited state evolution is considered, then from the MD equilibration step, we extract the last frame and consider it as input for the surface hopping ab initio MD, from which different fluorescent properties can be analyzed. The developed computational protocol can be applied for different probes, either in the ground state or excited states, and has been validated for different membrane phases.

### Probe Orientation as a Key Factor To Determine Membrane Phase: Diphenylhexatriene (DPH)

DPH is one of the most used probes for membrane studies, and its historical importance relies in rotational anisotropy analysis, because it can be considered as a rodlike molecule. Due to its extended π-conjugated chain between the two phenyl rings, several conformers can coexist for the same molecule. This parameter, essential to assess the correctness of the experimental rodlike model, is still challenging to observe, from a computational point of view, especially when the probe is embedded in an anisotropic environment like a lipid membrane, in which steric hindrance plays a decisive role in determining the natural presence of the different conformers. It should be noted that the approximation of DPH to a rod has its limitations since the angle between the transition dipole moment and the one of the approximating rod amounts up to 7°.^22^

By applying the computational methodology we have developed over the years, we were able to observe that different DPH conformers can be present in different membrane environments.^1^ From MD simulations of DPH embedded in three model membranes (DOPC, DPPC, and SM/Chol) representative of three different phases (Ld, So, and Lo, respectively), we observed that, in both Ld and So phases, only the all trans conformer is present, while Lo allows for large conformational changes due to the decreased steric hindrance, resulting in the presence of additional cis conformations. As a result, the different conformers affect the magnitude and orientation of the probe’s transition dipole moment in the different membranes. Moreover, the probe photoselection is heavily affected by the conformations due to different influences of the anisotropic environment, resulting in different optical response. It is worth noting here the link between the position and orientation of the probe in the membrane and its optical response. We know that the center of mass of the probe induces an increase of the distance from the membrane center going from DPPC (So) over DOPC (Ld) to SM/Chol (Lo).^22^ Thereupon, as DPH is vertically oriented compared to the membranes’ surfaces, one phenyl ring sticks out more toward the polar groups and the watery environment, while the other one is tucked deeper inside the aliphatic phase. The proximity to the membrane surface for SM/Chol is enough to allow for the presence of different conformers, while the dihedral angles, which are gradually located closer to the membrane center, remain indifferent to the characteristics of the lipid bilayer. Meanwhile, in DOPC and DPPC, the probe molecule remains rather rigid, it is flexible in SM/Chol, and polar groups are found in its close proximity.

**Figure 2:**
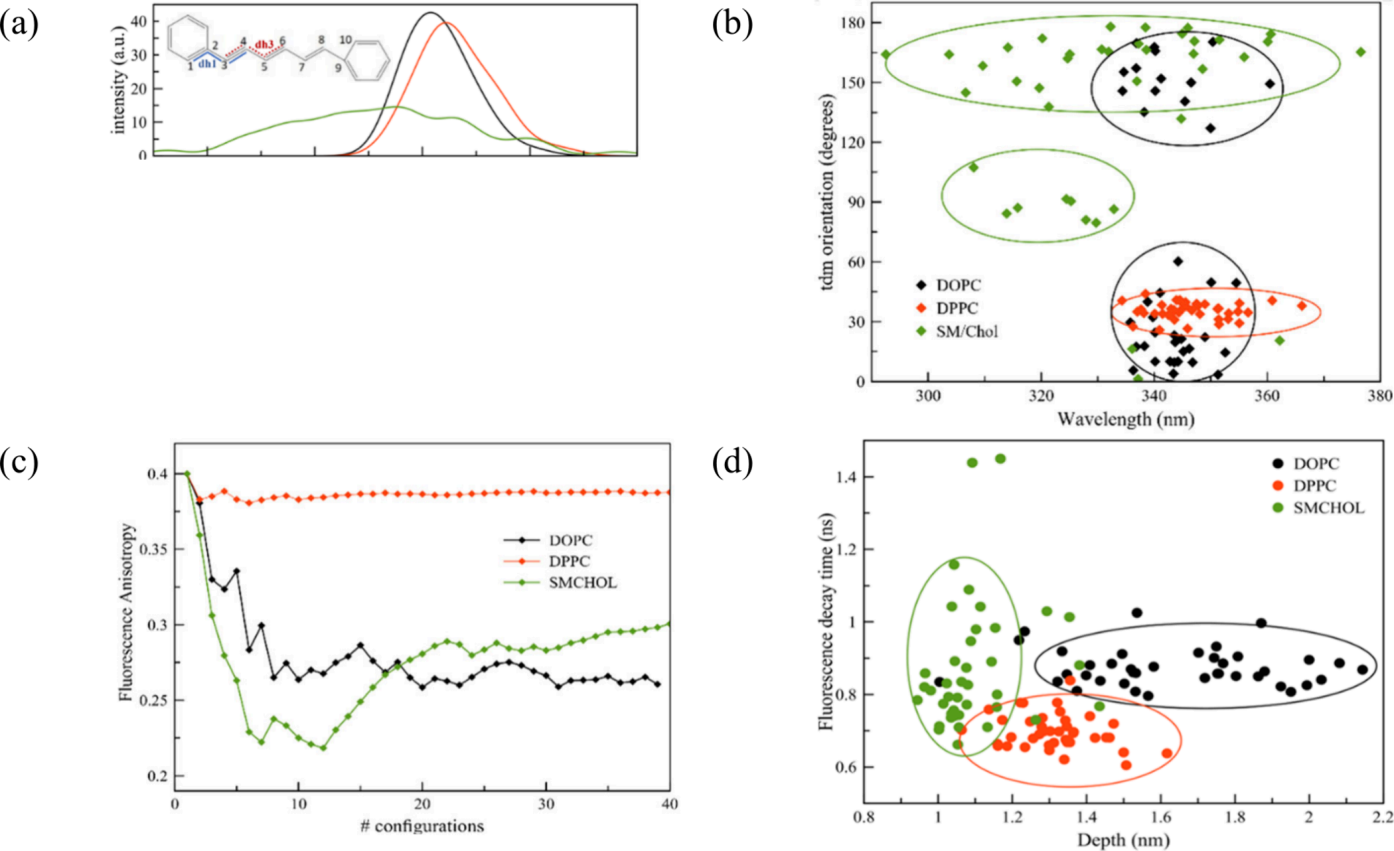
(a) For the three membranes, the absorption spectra of DPH are given along with (b) the correlation between the absorption wavelength and orientation of the transition dipole moment (tdm); (c) fluorescence anisotropy decay; (d) correlation between the fluorescence decay time and the location of the DPH probe. The difference between the P atoms at the membrane surface and the center of mass of DPH defines the depth. [Reproduced from ref (1). Available under a CC-BY license. Copyright 2020, Osella, S.; Paloncýová, M.; Sahi, M.; Knippenberg, S.]

The absorption spectra of DPH when embedded in DOPC and DPPC membranes show a high degree of similarity, although there is a minor red shift of 10 nm observed in DPPC (Figure 2a). Based on our MD analysis, it has been determined water is found at 1.3 and 0.9 nm away from the center of the membrane, respectively.^23^ This presence of water molecules at such distances could account for the observed red shift. Conversely, when the probe is placed in SM/Chol, its absorption spectra become significantly wider than those observed in the other two membrane types. This broadening is associated with the alignment of the transition dipole moment (tdm) of DPH, which is defined by the angle between the tdm vector and the membrane’s perpendicular axis. In the case of DPPC, a singular alignment is observed, which accounts for the spectrum’s single peak. However, in DOPC, there are two distinct tdm distributions, indicating a uniform orientation and hinting at the occurrence of what are known as flip-flops, a phenomenon that has been documented in prior studies.^1,22^ Therefore, both distributions occur within the same wavelength range, which clarifies why a single-peaked spectrum is observed. In contrast, a distinct pattern emerges when the probe is submerged in SM/Chol. Here, the tdm orientation exhibits a single distribution, yet it spans a broad spectrum of wavelengths (Figure 2b). This pattern is closely linked to the conformational changes previously mentioned.

As a result, both the probe’s varying depths and its specific alignment, relative to the membrane’s normal axis, cause significant variations in OPA for the Lo phase. This indicates that DPH is capable of distinguishing among the three phases discussed, causing a pronounced alteration in absorption spectral shape when it is incorporated into the Lo phase.

Given that fluorescence spectroscopy is a widely utilized method for analyzing lipid membranes, our computational approach also explored various fluorescence characteristics. Specifically, for DPH, we focused on fluorescence anisotropy analysis. This analysis is intimately connected to how freely the probe can rotate within the anisotropic environment of the membrane, reinforcing the observations previously discussed. As a general principle, when the probe’s rotation is restricted by its surroundings, anisotropy remains pronounced, and its reduction is minimized. Conversely, in lipid membranes that permit rotation, the opposite effect is observed. The observed anisotropy levels are influenced by the membrane phase in which DPH resides (Figure 2c). Notably, in DPPC, minimal anisotropy reduction is detected, aligning with expectations due to significant rotational constraints. However, a marked reduction in anisotropy is noted in the DOPC membrane. Previous studies have also highlighted distinct differences when analyzing the SM/Chol mixture; initially, anisotropy decreases sharply due to the DPH molecule’s semiflip and its perpendicular alignment to the membrane’s *z*-axis. Subsequently, anisotropy increases once more as the DPH probe aligns more parallell to the *z*-axis. To further understand these findings, we conducted additional analysis examining the relationship between decay time and DPH’s positioning within various membranes (Figure 2d). In both Ld and So phases, we observed a wide variance in the probe’s location, which did not match the variance in fluorescence decay time. Conversely, for the SM/Chol mixture, while the probe’s depth variation is minimal, the decay time shows a significant variance. This further analysis of decay times supports the conclusion that in DOPC and SM/Chol, the decay times are comparable, albeit for contrasting reasons.

### Laurdan: An Unexpected Molecular Rotor

In 2019, through multiscale modeling and simulations of time-dependent fluorescence anisotropy studies, we proposed that Laurdan, traditionally recognized as a solvatochromic probe, could also function as a molecular rotor.^2^ Early 2021, our finding was experimentally confirmed by Reinholdt.^24^ We note here that changes in the molecular rotor’s frictional resistance to rotational and translational motions alter the fluorescence lifetime. This results in significant variations in fluorescence quantum yield, depending on how easily the probe can rotate in different membrane phases.^25^

**Figure 3:**
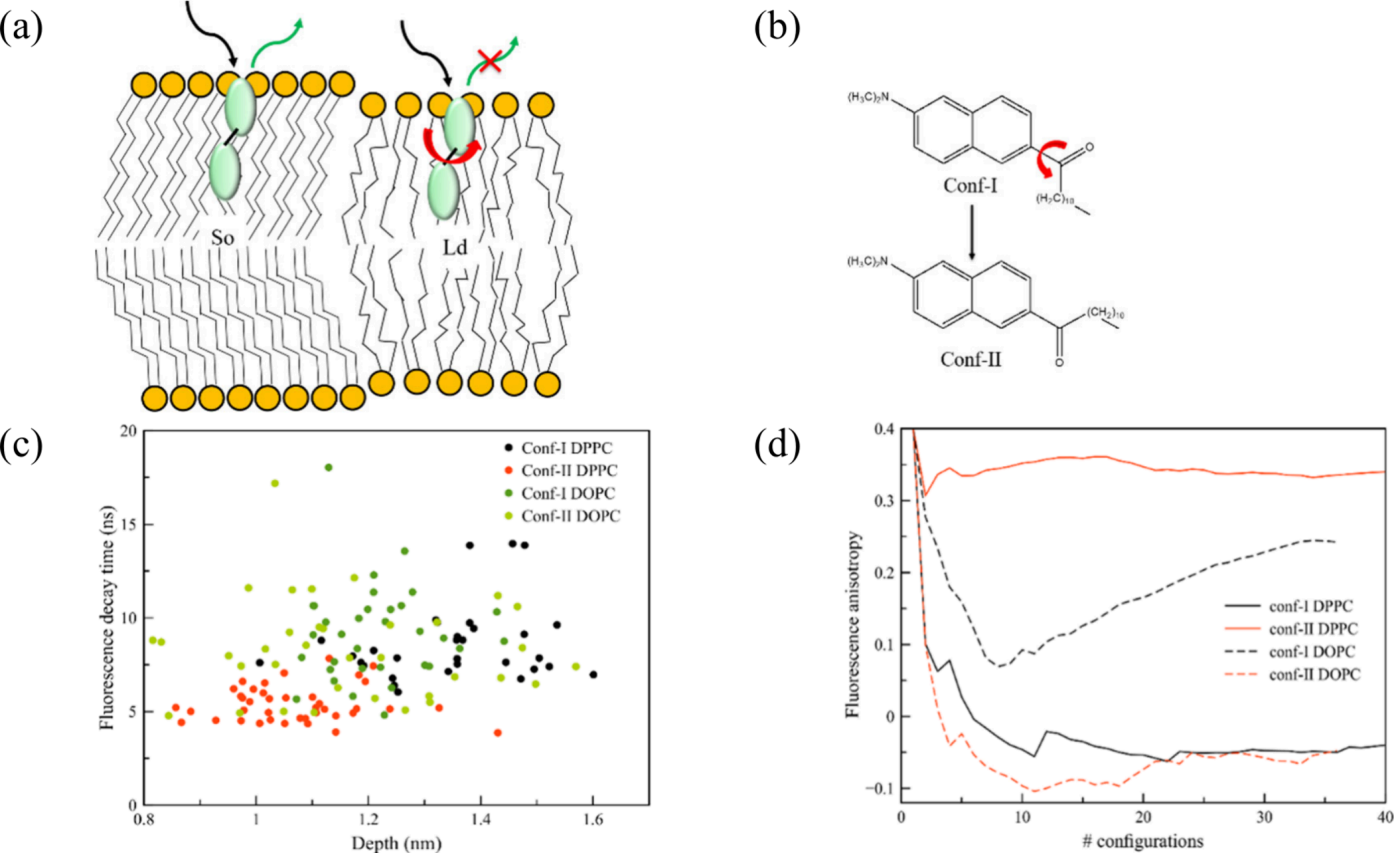
(a) Schematic representation of the interaction of a molecular rotor with different lipid bilayers, (b) the two conformations of Laurdan, (c) fluorescence decay time in function of the position of both conformers in DPPC (So) and DOPC (Ld), with each dot representing a snapshot from MD simulations, and (d) fluorescence anisotropy decay for the extracted MD snapshots of the two conformations in both DPPC (So) and DOPC (Ld) membranes. [Reproduced from ref (2). Copyright 2019, American Chemical Society, Washington, DC.]

We have demonstrated that Laurdan can be characterized by two distinct isomers, which partition differently in a So gel phase and an Ld phase, when considering two model membranes, DPPC and DOPC, respectively. It leads to different localizations and optical properties in the different environments (an overview can be found in Table 1).^26^ By applying our computational protocol, we saw the two conformers interchanging in the fluid DOPC (Ld) membrane. This process is fully obstructed in the more viscous DPPC (So) phase. This led us to suggest that, besides a solvatochromic probe, Laurdan can be seen as a molecular rotor. This should be directly related to the differences in optical properties. In fact, when embedded in DPPC (So), the high viscosity of the medium inhibits rotation, in which is relatively beneficial to the fluorescence quantum yield. Conversely, in DOPC (Ld), the quantum yield and lifetime should decrease because the free rotation allows for several nonradiative decay channels (Figures 3a and 3b).

**Table 1:** Elongated and Bent Realizations of Laurdan in DPPC (So and Ld Phases) and DOPC (Ld) in the Ground State (GS) and First Excited State (S1)

	So		Ld			
	DPPC		DPPC		DOPC	
	GS^a^	S1^b^	GS^a^	S1^b^	*GS*^a^^,^^c^	S1^a^^,^^c^
Conf-I	elongated	bent	bent	elongated	bent	bent
Conf-II	bent	elongated	elongated	elongated	bent	bent

a Data taken from refs (2) and (29).

b Data taken from Knippenberg *et al.*, Cells 2024, *13*, 1232.

c Data taken from ref (27).

These structural differences between the two conformers of Laurdan are subtle and difficult to evaluate experimentally. However, hybrid QM/MM calculations can be used to enable and propose probes at a relatively low cost and within limited time frames. This approach allows for a comprehensive study of many commonly investigated linear and nonlinear optical properties, as well as hyperpolarizability response and fluorescence analysis. If differences in optical properties are observed due to the presence of two distinct conformations, we can conclude that Laurdan resides in a DPPC (So) membrane. Conversely, if the optical response is the same for both conformations, the probe is more likely embedded in a DOPC (Ld) phase and can be described as a molecular rotor. Additionally, the lipid bilayer induces varying degrees of steric hindrance, which results into a broadening of the absorption to longer wavelengths. This broadening can be directly related to the different degrees of rotational freedom of the transition dipole moment of the probe in vacuum, compared to within the membranes.

Our results confirm this assumption. The distinct orientations of the conformers (specifically the orientation of the carbonyl group) and the significant hindrance experienced when inserted into the DPPC (So) membrane are reflected in a pronounced bathochromic shift observed for Conf-II, compared to Conf-I. Conversely, in DOPC (Ld), the two conformers can interconvert,^27,28^ resulting in negligible differences in the absorption spectra. Additionally, when the probes are inserted into a DOPC membrane in the Ld phase the detailed structure in the absorption spectra observed for DPPC (So) disappears. Thus, the differences observed in different membranes are primarily due to changes in position and orientation, with respect to the hydrophobic lipid tails, the high headgroup density region, or the water layer. The eventual differences in absorption spectra due to conformational changes strongly indicates the versatility of the Laurdan probe. This versatility has been recently shown to be crucial in explaining observations in generalized polarization and time-resolved fluorescence experiments in large unilamellar vesicles.^29^

To evaluate Laurdan as a molecular rotor, fluorescence studies should be performed. The fluorescence intensity, lifetime, or anisotropy between the two different conformations should vary in different environments due to differing fluidity. Among these, fluorescence lifetime is probably the easiest to assess through both computation and experiments. Fluorescence lifetime can be significantly affected by changes in fluorophore conformation, quenching, and fluctuations in dielectric properties from the surrounding environment when a probe is embedded in a biological context. This decay time can be represented as a function of the depths of both probes within the DPPC and DOPC membranes (Figure 3c). From this plot, we can clearly observe two populations, with a notable difference in position between the two probes in DPPC (So). The data for Conf-II show slightly shorter lifetimes compared to Conf-I, despite the substantial spread of data points along the vertical axis of decay time. However, for DOPC (Ld), the separation between data points for both conformers is negligible. Furthermore, fluorescence anisotropy supports this perspective (Figure 3d), as the environmental impact on fluorescence depolarization for the two conformers is distinctly observable. In DPPC (So), fluorescence is depolarized for Conf-I, whereas it does not affect the emission from Conf-II. Consequently, similar to a “’classical” molecular rotor, the combination of medium viscosity and restricted conversion between conformations results in distinctive fluorescence signals and suppression of anisotropy decay for Conf-II. However, in DOPC (Ld), where interconversion between the two conformations is allowed, depolarization of emission is evident, and the solvatochromic character predominates.

### A Promising Photoswitch for Phase Detection: Azobenzene

Azobenzene is a prototype for a molecular machine, capable of switching between two (meta)stable states (trans and cis) in a controlled manner using light as the sole energy source. By harnessing this property and applying it to a biological environment such as a lipid membrane, we can create an “on/off” photoswitch where one state is active and the other is inactive. If these states exhibit different activity levels in different membrane phases, we achieve a multifunctional probe. Our computational analysis showed that the position and orientation of a cis azobenzene derivative in an Ld (DOPC) membrane differ markedly from those in a So (DPPC) phase, making this probe a significant on/off switch for membrane classification.^30^

**Figure 4:**
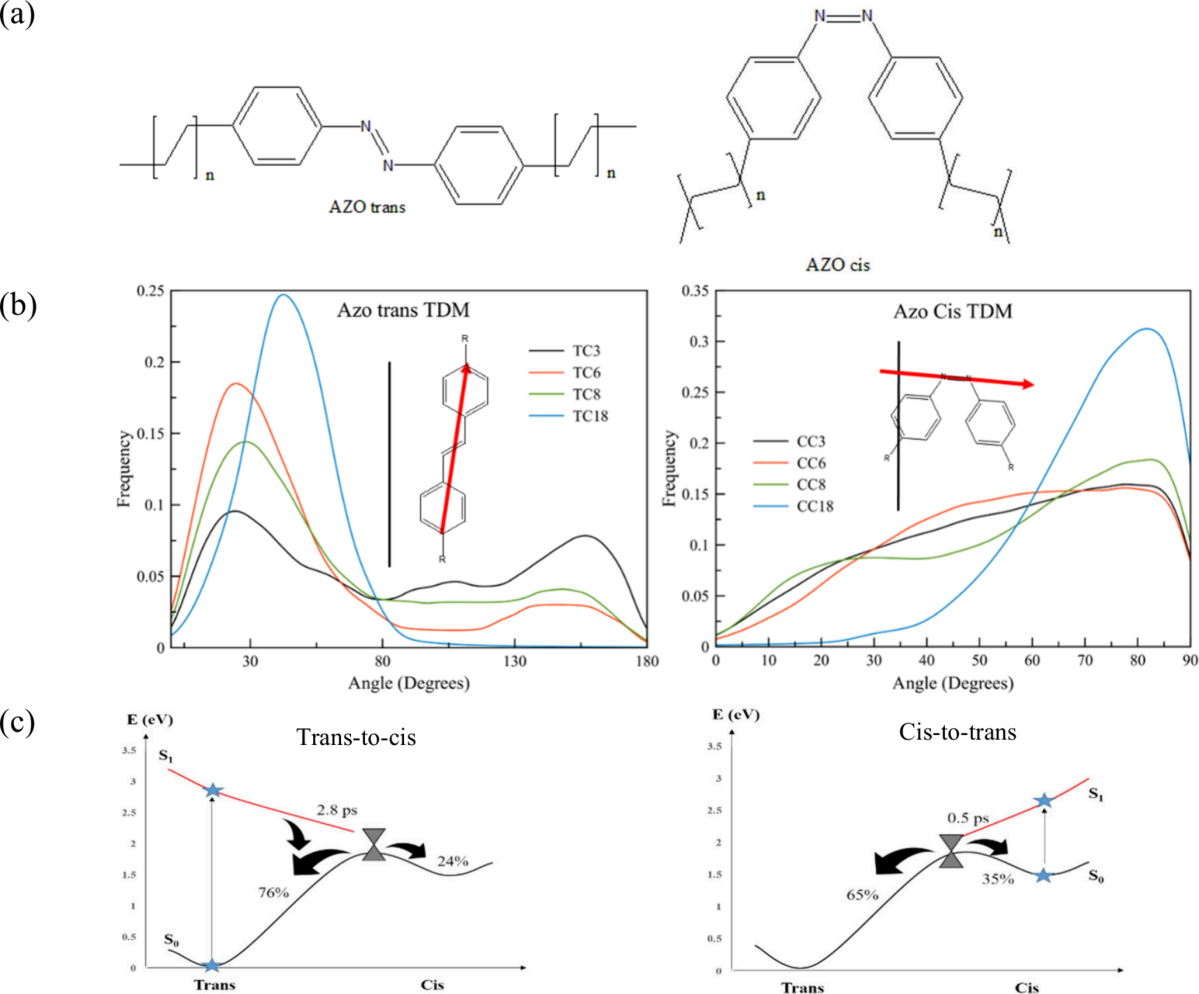
(a) Molecular structures of the studied trans and cis azobenzene isomers (*n* = 2, 5, 7, 17). (b) Distribution of the angle between the transition dipole moment of the probe and the normal to the membrane surface for both isomers. The insets show the angle between the tdm vector (in red) and the bilayer normal (vertical black line). (c) Sketch of the photoisomerization of the azobenzene probe put in a DPPC lipid membrane at 323 K for the trans-to-cis pathway and for the cis-to-trans pathway. [Reproduced from refs (3) (Copyright 2020, American Chemical Society, Washington, DC) and (4) (available under a CC-BY license, Copyright 2023, Osella, S.; Granucci, G.; Persico, M.; Knippenberg, S.).]

To expand the study, we also considered an azobenzene with varying lengths of four symmetric alkyl tails (Figure 4a) and assess their influence not only on the location of the probe in the membrane, but also on the photoswitching ability while increasing the tails length.^3^

We notice a consistent trend where the trans isomer gradually moves toward the center of the lipid bilayer as the length of its tails increases, whereas the cis isomer remains near the membrane surface. This phenomenon can be understood by examining the dipole moments of the two isomers; specifically, the less-polar trans isomer is drawn toward the bilayer’s center to enhance lipophilic interactions, while the tails of the more polar cis isomer drive it closer to the membrane surface to optimize hydrophilic interactions. This might seem unexpected, given that the probe is in a liquid disordered phase which should, theoretically, permit extensive tail movement. However, the interplay of the tails’ lengths, steric hindrance, and push/pull effects, prevents the trans isomer from adopting a more parallel alignment with the membrane surface, an alignment that the cis isomer can achieve. This observation is supported by the probes’ orientations, measured by the angle between the transition dipole moment (tdm) and the membrane’s perpendicular axis (as shown in Figure 4b). Here, the influence of increasing tail length becomes more apparent. For the trans isomers, the interaction between the tails and the lipids is initially minimal. But as we move to longer tails in the series, the pulling effect of the tails becomes dominant, leading to an orientation that increasingly aligns parallel to the membrane’s perpendicular axis. The reverse is observed for the cis isomer, where even the shortest tails result in a more perpendicular orientation to the bilayer’s axis, and the molecule’s wobbling decreases as the tails reach their maximum length, which almost results in a perpendicular alignment of the entire probe to the normal of the membrane, highlighting the distinct push/pull effects of the tails on the two isomers.

This has significant implications for optical properties, especially absorption, as the environmental-induced twist in the structure increases the transition dipole moment from the first excited state, making the transition allowed for both isomers. Additionally, the number of absorption peaks grows with tail length, indicating greater conformational changes due to both isomers’ fluctuations within the membrane.

Motivated by these findings, we advanced our research to explore the trans–cis photoisomerization of the azobenzene probe within a DPPC (So) membrane through ab initio molecular dynamic–surface hopping simulations, aiming to understand the mechanism behind this dynamic process.^4^ Our findings indicate that, beyond the influence of a biological setting, the differences in potential energy surface of the two isomers significantly contribute to the underlying mechanisms. Specifically, when examining the S_1_ potential energy in relation to the CNNC torsion angle, we note a steep curve for the cis-to-trans isomerization (as depicted in Figure 4c), which creates a stronger driving force for the cis form, enabling it to overcome environmental barriers. Conversely, the more gradual slope of the trans isomer’s energy surface renders it more susceptible to steric hindrances from the membrane, leading to a considerably slower trans-to-cis isomerization process on the picosecond time scale. In contrast, the cis-to-trans isomerization occurs almost instantaneously (within <0.5 ps), which is a key factor behind the lower photoisomerization efficiency of 24% for the trans-to-cis transition, as opposed to a significantly higher efficiency of 65% for the cis-to-trans conversion. These observations strongly support the conclusion that a torsional mechanism predominates in the trans-to-cis transition, whereas a “pedal-like” or Hula mechanism is more evident in the cis-to-trans isomerization.

The different responses of the two isomers are further visible in their fluorescence anisotropy decay patterns. Transitioning from trans to cis, the decay in fluorescence anisotropy is minimal, whereas a more comprehensive decay is noted when moving from cis to trans, attributed to the rapid shift in the orientation of the tdm. This results in a pronounced difference in behavior between the two isomers when they are incorporated into DPPC, making the cis isomer the “active” state. Concurrently, this enhances the utility of azobenzene as a fluorescent probe for identifying membrane phases.

## Conclusions and Perspectives

In the studies we have presented, utilizing a thorough computational approach that integrates hybrid quantum mechanics/molecular mechanics (QM/MM) techniques, we have elucidated the relationship between the positioning and optical characteristics of various fluorescent probes within different phases of membranes.

We have demonstrated that DPH can serve as an effective probe for identifying membrane phases when viewed as a chromophore. Employing both linear optical and fluorescence methodologies allows for the distinct recognition of membrane phases, setting the stage for DPH’s broad application as a discriminating probe. Furthermore, our findings suggest that Laurdan should also be regarded as a molecular rotor, given the presence of two distinct conformers across different membrane phases. Our simulations indicate a significant variance in fluorescence anisotropy decay between these conformers in a viscous environment like DPPC (So), which should be observed from experiments.

Additionally, our research proposes azobenzene as a potentially universal probe for phase detection. The combination of simulations with experimental investigations into azobenzene’s photoisomerization in DPPC lipid bilayers can initiate applications in fluorescence and bioimaging fields. Given that this work represents one of the initial forays into (simulating) azobenzene probes within lipid bilayers, to our knowledge, we encourage groups focused on experimental work to validate and eventually extend our findings. Moreover, considering the significant role of the push/pull effect on the NLO properties, it warrants attention in the future development of photosensitive probes for membrane recognition.

Toward the future, ab initio molecular dynamics methods and surface hopping schemes should be applied to lipid membranes and widely known embedded probes like Laurdan and DPH, for which we pointed at the importance of the conformational changes to interpret experimental Fluorescence Lifetime IMages (FLIMs) data.

A further step toward accuracy of our protocol is the inclusion of polarizable force fields in the description of the system, as they capture induction effects of the solvent toward the probe and vice versa. This will have a double benefit of a better description of the MD simulation allowing for water polarization (important when the Lo phase is considered) but also will improve the QM/MM part, as a polarizable embedding could be used, enhancing the accuracy of the optical properties’ description. Following this approach, the long-standing debate in the literature focusing on the nature of Laurdan’s first excited state can be solved. Until now, many works focused on Gromos force fields, which were the first ones to parametrize saturated lipids and which ensured a wide consistency of the presented data. For future work on lipid bilayers, force fields with increased flexibility in the description of sterol molecules and water environments should be considered.^31^ An additional step toward higher accuracy for our computational protocol is the use of machine-learned force fields (MLFFs), trained on ab initio MD data, which start to become of use also when describing biological systems. By using tens of thousands of frames in combination with neural networks (QUIP, DeePMD, NNP-MM, to name a few) and runs at different temperatures, parameters can be obtained to get access to stable excited-state molecular-dynamics runs. By reverting to artificial intelligence and standard techniques like random forest, the characteristics of the probes can be predicted in complex compositions, multiphasic systems, and biological environments constituting out of those different lipids and lipid regions. Curvature effects can be taken into account.^32^ Photoinduced stimuli might also affect transport, permeability, and regulatory effects of membranes as these are exerted through receptors, dedicated membrane proteins, and channels,^33^ which are finally of importance in nonlinear optogenetics.^34,35^ It can be remarked however that the analysis of fluorescence experiments in complex media is only possible when the influence of the different components is known.

The computational protocols reported here can be combined with single-molecule fluorescence resonance energy transfer microscopy^36^ and permit one to gain insight into destabilizing conditions and mutations which rather result in unfolding.^37^ The same techniques can be used to investigate broader soft-matter problems, which incorporate polymers, glues, and salts, as here also phase transitions, transport, and permeability issues are encountered.^38,39^ Their temporal evolutions and relaxation times can all be investigated using multiscale modeling techniques, autocorrelation functions, and anisotropy techniques.^40^

We encourage experimentalists and, more specifically, those who work with single molecule spectroscopy, fluorescence correlation spectroscopy, and quantitative microscopy imaging techniques to validate our results and apply them in biomedical settings where cancerous disorders are preluded by aberrant cell membranes.^41−44^
